# Consequences of kelp forest ecosystem shifts and predictors of persistence through multiple stressors

**DOI:** 10.1098/rspb.2023.2749

**Published:** 2024-02-07

**Authors:** Joshua G. Smith, Daniel Malone, Mark H. Carr

**Affiliations:** ^1^ Conservation and Science Division, Monterey Bay Aquarium, Monterey, CA, USA; ^2^ Department of Ecology and Evolutionary Biology, University of California Santa Cruz, Santa Cruz, CA, USA

**Keywords:** community structure, sea urchin, climate change, regime shift, alternative stable states, marine heatwave

## Abstract

Ecological communities can be stable over multiple generations, or rapidly shift into structurally and functionally different configurations. In kelp forest ecosystems, overgrazing by sea urchins can abruptly shift forests into alternative states that are void of macroalgae and primarily dominated by actively grazing sea urchins. Beginning in 2014, a sea urchin outbreak along the central coast of California resulted in a patchy mosaic of remnant forests interspersed with sea urchin barrens. In this study, we used a 14-year subtidal monitoring dataset of invertebrates, algae, and fishes to explore changes in community structure associated with the loss of forests. We found that the spatial mosaic of barrens and forests resulted in a region-wide shift in community structure. However, the magnitude of kelp forest loss and taxonomic-level consequences were spatially heterogeneous. Taxonomic diversity declined across the region, but there were no declines in richness for any group, suggesting compositional redistribution. Baseline ecological and environmental conditions, and sea urchin behaviour, explained the persistence of forests through multiple stressors. These results indicate that spatial heterogeneity in preexisting ecological and environmental conditions can explain patterns of community change.

## 1. Introduction

Ecological communities can be stable over multiple generations, or rapidly shift into structurally and functionally different configurations [1–4]. One of the most pressing challenges in the field of ecology is to identify and understand the mechanisms that buffer systems from change (i.e. resistance), or that enhance the ability for a system to return from a perturbation (i.e. resilience), and that drive alternations between states [5,6]. However, the capacity to explain and predict when, where, and under what conditions ecosystems are subject to change requires understanding how and why the structural integrity (i.e. species composition, diversity, interactions, trophic structure) of ecological communities varies across time and space [6–8].

State shifts in both terrestrial and marine environments can markedly alter the structure and functioning of ecosystems and can impart rapid changes to ecosystem services [4,9,10]. However, theoretical and empirical advances on the processes (i.e. environmental or biotic) that initiate state shifts are often constrained to opportunistic events in space and time that expose the boundaries at which shifts occur [3,11]. Events (either punctuated or continuous) that erode persistent community configurations may elucidate causal mechanisms that facilitate state shifts, including factors that reduce resilience, and the ecosystem-wide consequences that follow destabilization [4,11,12]. Like many terrestrial and aquatic ecosystems, herbivore outbreaks (i.e. marked shift in foraging behaviour) in coastal marine ecosystems can drive population and community dynamics that scale-up to influence ecosystem stability and resilience [13–16].

Separate from understanding the susceptibility of ecosystems to state shifts is the importance of understanding the ecological consequences of community restructuring. In coral reef, rocky intertidal and kelp forest ecosystems around the world, outbreaks in populations of herbivorous sea urchins have led to abrupt state transitions from macroalgae dominated communities to alternative sea urchin ‘barrens’ [17–20]. Sea urchin barrens are characterized by an abundance of exposed actively foraging sea urchins, are primarily dominated by encrusting red and coralline algae, and are devoid of macroalgae [19]. Barrens are generally highly unproductive and can persist for several years because of the ability of sea urchins to survive with minimal resources and consume newly recruited algae [21–23]. As such, sea urchin barrens are often considered a stable alternative ecosystem state because of intrinsic feedback mechanisms (e.g. positive sea urchin settlement reinforcement) that promote the persistence of that particular community configuration [18–20].

Although numerous studies have explored changes in algae, invertebrate and fish assemblages independently between kelp forest and sea urchin barren habitats [13,24,25], fewer have tracked entire community and ecosystem-level responses through the formation, expansion and persistence of sea urchin barrens over time. Such long-term studies are important for disentangling the spatial and temporal scales over which state shifts occur, including identifying the relative contributions of individual species responsible for community destabilization, and estimating the magnitude to which changes in community structure permeate entire trophic networks [26,27].

In 2014, kelp forests along the west coast of North America experienced a rapid and pronounced shift from highly expansive forests to large swaths of unproductive sea urchin barrens [28–30]. Of particular concern is whether (and how) this widespread kelp deforestation resulted in a marked shift in the predominant source of primary production (from macroalgae to plankton) and decreased food web complexity. Recent studies have identified considerable geographical variation in species responses and key functional groups to a marine heatwave and decline in kelp, most notably in Mexico and northern California kelp forests, where the extent of forest loss was region-wide [28,29]. In contrast to these region-wide shifts in system state, forest loss in central California was spatially heterogeneous resulting in mosaics of forests and barrens [30]. These mosaics allow for concurrent comparison of community structure in forest and barrens subjected to similar past and present environmental (oceanographic, geomorphological) conditions.

Here we examine the community-wide consequences of kelp deforestation along the central coast of California, where outbreaks of purple sea urchin (*Strongylocentrotus purpuratus*) grazers shifted a once expansive kelp forest to a mosaic landscape of sea urchin barrens interspersed with remnant patches of kelp [30]. The purpose of this study was to explore changes in community structure associated with the loss of forests as a result of overgrazing by sea urchins to test the following hypotheses: (1) the spatial mosaic of barrens and forests resulted in a region-wide shift in community structure relative to the years preceding the formation of the mosaic, (2) local change in community structure depends on whether forests persisted or transitioned to barrens, (3) community structure dynamics were spatially cohesive among sites that transitioned or persisted, and (4) the relative resistance (conversely, vulnerability) of persistent forests is explained by preexisting environmental and ecological conditions.

## 2. Methods

Using the mosaic landscape of sea urchin barrens interspersed with remnant patches of kelp forests, and a 14-year kelp forest community monitoring dataset that spanned the 2014 shift in forest states, we evaluated the environmental and ecological correlates with resistance (conversely, vulnerability) to state transitions, and the consequences of sea urchin grazing and forest loss on taxonomic community structure within and across sites of diverging ecosystem states.

### (a) Study area

**Figure 1.**
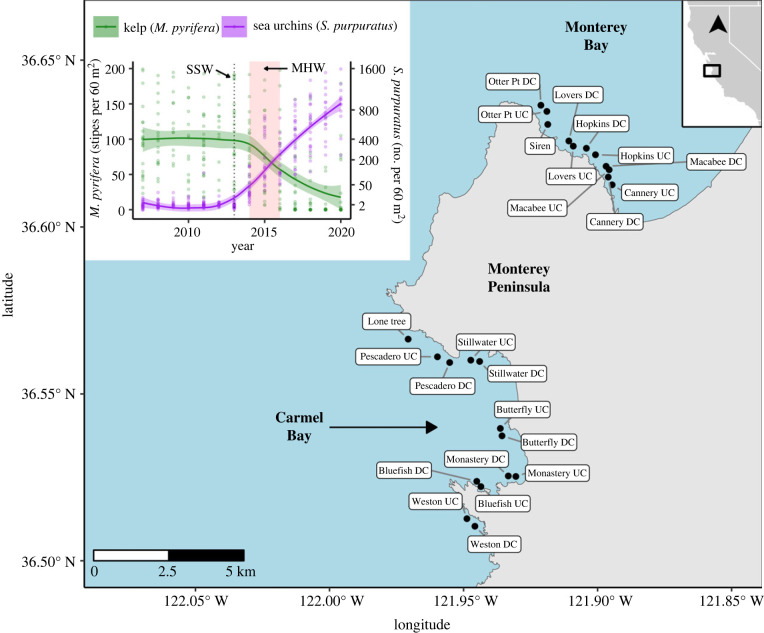
Study area along the Monterey Peninsula, California, USA. Black points on the map depict the locations of 24 long-term subtidal monitoring sites [31]. The inset figure depicts temporal trends of kelp (*Macrocystis pyrifera*) stipe density (left vertical axis) and purple sea urchin (*Strongylocentrotus purpuratus*) density (right vertical axis) across all 24 sites. Each point in the inset figure represents the mean stipe (green) or sea urchin (purple) density at a site. Splines (λ = 0.05) were fitted across interannual means with 95% confidence intervals. The vertical dotted line represents the timing of the 2013 sea star wasting (SSW) event, and the 2014–2016 marine heatwave (MHW) is shaded in red.

This study was conducted along the northern coast of the Monterey Peninsula and in Carmel Bay, California, USA (figure 1). The study region is located where the geographical range of two canopy-forming kelps, the giant kelp (*Macrocystis pyrifera*) and the bull kelp (*Nereocystis luetkeana*), overlap along the west coast of North America [32]. However, around the Monterey Peninsula, giant kelp is the historically predominant kelp species. With three exceptions (Lone tree, Pescadero UC, Pescadero DC; figure 1), all sites included in this study are located within marine protected areas (full protection established in 2007) that prohibit the take of marine invertebrates and algae. As an eastern boundary system, coastal upwelling and wave disturbance are the predominant drivers of kelp productivity on both sides of the Monterey Peninsula [33]. However, considerable heterogeneity in mixing results from variation in bathymetric features and wind forcing on either side of the peninsula [34]. Sites around the peninsula also vary markedly in wave exposure [35]. In 2014, active grazing by purple sea urchins (*Strongylocentrotus purpuratus*) shifted forests on both the north side of the Monterey Peninsula and Carmel Bay to a patchy mosaic of remnant kelp forests interspersed with sea urchin barrens that are void of macroalgae [36]. The sea urchin outbreak occurred shortly after a 2013 catastrophic sea star epizootic, and coincided with the onset of the 2014–2016 Northeastern Pacific Marine heatwave [30,37,38].

### (b) Kelp forest monitoring surveys

To characterize the spatial and temporal patterns of community structure, we used long-term subtidal monitoring data collected by the Partnership for Interdisciplinary Studies of Coastal Oceans (PISCO) [31]. Although PISCO sampling began in 1999, we elected to use 2007–2020 as our study period to standardize relatively equal sampling effort among sites, years, and before and after the 2014–2016 marine heatwave (i.e. fewer sites were sampled prior to 2007).

The PISCO subtidal sampling design and protocols are described in detail in Malone *et al*. [31]. Briefly, we focused our analyses on 24 PISCO sites surveyed annually between mid-June to mid-October from 2007–2020 in Carmel Bay and southern Monterey Bay, California (figure 1; electronic supplementary material, figure S1 and table S1). Sites are identified by permanent GPS coordinates, and divers use compass headings and stratified isobaths to demarcate the position of transects for consistent sampling. Annual surveys at each site consist of visual surveys by SCUBA divers of the density and percentage cover of conspicuous benthic algae, invertebrates, and benthic and water column-dwelling fishes. Density and percentage cover estimates of conspicuous benthic algae and invertebrates are recorded along six replicate 2 m × 30 m transects stratified across three bottom depths (5 m, 12.5 m, 20 m; two transects per depth level). Densities of mobile and individually distinguishable sessile invertebrates and stipitate brown algae (order Laminariales) are identified to the species level. Sessile macro-invertebrates and other macroalgae difficult to distinguish individually (e.g. colonial sponges, tunicates, foliose algae) are quantified using uniform point-contact (UPC) estimates of percentage cover every metre along each 30 m long transect (30 total points per transect). Colonial invertebrates (e.g. sponges, tunicates, bryozoans) surveyed along these UPC transects are identified to the Phylum (e.g. Porifera, Bryozoa) level and macroalgae are grouped into morphologically distinct categories (electronic supplementary material, table S2 for full taxonomic list). Fish densities (number per reef area) are estimated along 12 replicate transects stratified across bottom depths (5 m, 10 m, 15 m, 20 m; three transects per depth level) and identified to the species level. Each fish transect consists of paired 2 m × 2 m × 30 m bottom and water column transects. For all analyses, we used the lowest taxonomic resolution possible across survey methods (electronic supplementary material, Methods).

### (c) Region-wide shift in community structure

We used a series of multivariate analyses to test the hypothesis that the spatial mosaic of barrens and forests resulted in a regional shift in taxonomic community structure (invertebrates, algae, fishes) relative to the years preceding the formation of the mosaic. First, we normalized (converted to *z*-scores) counts or percentage cover of each taxa across the three survey methods (swath, UPC, fish). This approach yields a scaled metric that has identical units (standard deviations) and a similar value range for all taxa regardless of original units (e.g. counts or percentage cover) and therefore allows for broad integration of survey methods to compare whole community-level dynamics among sites and years. This approach also results in taxa all having the same potential impact in the multivariate analyses, regardless of whether they are rare or common (we consider abundance in separate analyses described below). While biomass is typically used as a common currency across taxa [39], it is not suitable for our multivariate analyses because of the large number of kelp forest taxa that are difficult to accurately assign biomass estimates. Therefore, we elected to use a z-score normalization of the datasets to compare broad structural changes in taxonomic community structure.

To visualize community structure changes, we calculated a Bray–Curtis distance matrix (hereafter, ‘base matrix’) with a Hellinger transformation for all sites across all years using the *Vegan* package in R [40]. Using this base matrix, we plotted the centroids for each year (representative of all sites) using non-metric multidimensional scaling (nMDS) to examine community structure trajectories across the entire sampled region through time and in multivariate space. We then used a *k*-means cluster analysis and elbow plots to identify significant temporal groupings among the centroids. We used the results of this analysis to inform two distinct temporal clusters: 2007–2013 and 2014–2020. We then used a permutational analysis of variance (PERMANOVA) to test whether community structure significantly changed across the two clusters (from before to after the sea urchin outbreak). A single PERMANOVA was performed at the regional level (i.e. sites as replicates) and for each independent site using 999 permutation-based resampling.

In addition to evaluating the region-wide community structure changes, we also assessed temporal variation in taxonomic diversity (number of taxa and their evenness), richness (number of taxa), and evenness (relative abundance of taxa). We used raw count (frequency) data by survey method (fish, kelp and mobile inverts, and macroalgae and sessile inverts), rather than the normalized dataset used in the community structure analyses, since the normalization process diminishes the influence of frequency. Shannon diversity, taxonomic richness, and evenness were calculated at the site level using the mean relative abundance of taxa across replicate transects for a given site.

### (d) Spatial cohesion of community structure

To determine whether particular spatial patterns and scale of community change emerged across the mosaic, we evaluated the cohesion of community structure trajectory among all 24 sampled locations. First, for each site, we compared the annual multivariate distance between a given year and the ‘before’ (2007–2013) site-level centroid. Multivariate distance was calculated using the betadisper function in *Vegan*, which reduces the base matrix to principal coordinates, allowing for estimation of multivariate distance. We also explored whether individual sites moved synchronously through multivariate space, or if some sites were asynchronous over time, and whether there was any spatial pattern to observed synchrony. For this analysis, we used a Procrustes test to examine similarity among the site-level ordinated data. The Procrustes analysis determines the optimal alignment between two ordinated objects (sites) through superimposition, which involves translating the centroid of one configuration to the origin of the coordinate system, rotating both configurations to minimize the differences, and scaling one configuration to match the other in terms of size. Once the configurations are aligned, a similarity statistic and correlation coefficient are generated that describe the overall fit (similarity) between the two centroid trajectories. This analysis was performed pairwise for all site combinations to assess cohesion. However, because the Procrustes analysis requires balanced samples (equal number of years between pairwise site comparisons), we used linear interpolation for sites with incomplete time series (electronic supplementary material, figure S1).

### (e) Functional traits and taxonomic variation

We fit a series of generalized linear models to multivariate abundance data to explore the interactive effect of functional traits and period (2007–2013 versus 2014–2020) on the relative abundance of taxa. These models were constructed separately for fishes, and for invertebrates and macroalgae (based on sampling methods), using the *mvabund* package in R. All taxa were assigned to one of the following functional traits using a published trait table [31]: detritivore, macroalgae, planktivore, herbivore, microinvertivore, macroinvertivore or piscivore. Each model used a negative binomial distribution with a least absolute shrinkage and selection operator (LASSO) penalty.

To evaluate the relative change and contribution of taxa to overall shifts in community structure, we compared the relative change in the abundance of taxa between persistent forests and forests that became barrens (hereafter, ‘transitioned’ sites). Importantly, we consider persistent forests as locations where kelp density did not significantly decline after the marine heatwave, regardless of any potential changes in community structure. Giant kelp (*Macrocystis pyrifera*) is the dominant habitat-forming species in this system, and therefore we were interested in exploring whether the persistence of this foundation species (*sensu* [41]) resulted in fewer declines in the abundance of other taxa relative to sites that experienced forest loss.

Persistent forests were identified using a *t*-test on the mean kelp density (stipes per 60 m^2^) at each site before (2007–2013) versus after (2017–2020) the marine heatwave. Heatwave years (2014–2016) were not included in this evaluation because of potential lagged effects (e.g. including heatwave years in the ‘before’ or ‘after’ grouping may confound the results because kelp loss may occur the following year). Persistent forests were identified by a non-significant test statistic, and significance indicated that forest density declined in the post-heatwave period relative to the pre-heatwave period.

We used the *mvabund* package in R to identify taxa that significantly explained differences in persistent and transitioned forests across the two focal time periods (before versus after the heatwave [42]). The *mvabund* package uses fitted simultaneous generalized linear models to account for nonlinear mean to variance relationships, allowing for model-based comparisons of individual taxa between groups. This analysis was performed separately for persistent and transitioned forests using the site-level mean counts of taxa. A univariate analysis of variance (ANOVA) test was then performed to evaluate changes in multiple taxa simultaneously. The resulting output provides a summary table that presents the results of the ANOVA tests for each taxa within persistent and transitioned forests. We then mapped significant taxa (i.e. those that explained community changes) back to the raw data and evaluated changes in absolute abundance before versus after 2014. The final results of this approach yield the number and identity of taxa that define community changes and their absolute change in abundance (positive or negative). We then inferred the consequences of forest loss on taxon-level changes by comparatively evaluating the number, identity and percentage abundance change between persistent and transitioned forests.

### (f) Environmental and ecological predictors of kelp persistence

We explored whether kelp persistence (conversely, vulnerability) was explained or could be predicted by environmental variables and ecological attributes. This analysis included the baseline (from 2007–2013) mean kelp density and coefficient of variation (as a measure of kelp stability), annual site-level sea urchin density and simulated behaviour (electronic supplementary material, Methods), sea surface temperature, net primary productivity, upwelling intensity, seafloor rugosity, wave orbital velocity, depth, reef slope and wave height (electronic supplementary material, table S1). We inferred sea urchin behaviour (proportion of actively grazing sea urchins) using observed stipe density and published data on the relationship between standing kelp stipe density and the proportion of actively grazing (versus passive) sea urchins (electronic supplementary material, Methods) [36]. Seafloor rugosity (vector ruggedness) and slope were obtained directly from the California Seafloor Mapping Program [43] and averaged at a 2 m resolution for each site. These multibeam bathymetry data were also used to estimate the average depth (m) at each site. Sea surface temperature (SST, °C) was calculated at 1 km daily resolution from MURSST [44] and averaged for each month and year. We also obtained estimates of the Biologically Effective Upwelling Transport Index (BEUTI) calculated in 1 degree latitude bins [45], and a retrogressive Net Primary Productivity (NPP) product (electronic supplementary material, table S1).

We used a generalized additive model (GAM) to identify the relative correlative strength of putative explanatory variables of kelp density over the course of the study period (2007–2020) across all 24 long-term monitoring sites. The full model included annual estimates of kelp stipe density as a function of each ecological and environmental variable. All predictors were included as annual smoothing terms, and year was added as a cyclic cubic regression spline to account for periodic trends over time in the data. We used a restricted maximum-likelihood profile and cubic spline to determine the optimal level of smoothing for each predictor. The best fit model was identified using shrinkage term selection [46] to identify the most parsimonious predictors of kelp density.

## 3. Results

**Figure 2.**
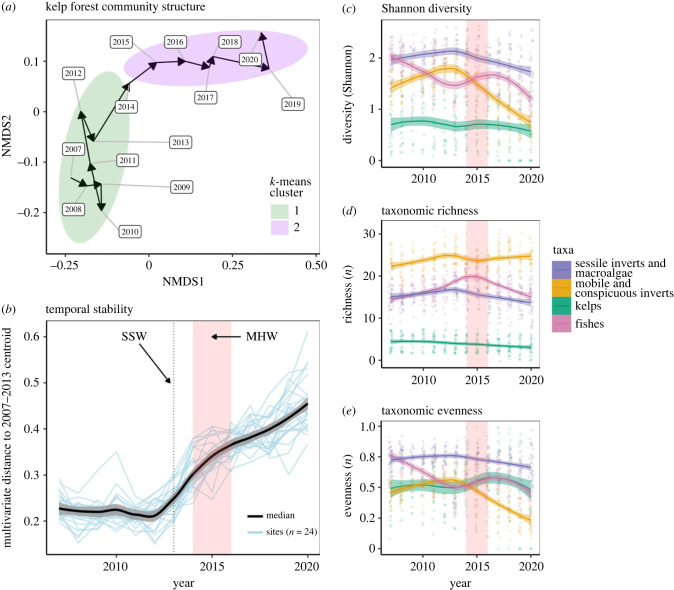
Regional community metrics over time. (*a*) Changes in community structure using a 2D nMDS plot of centroids across all 24 sampled locations. Each point represents the centroid for a given year connected with interannual arrows. The green and purple ellipses depict significantly different clusters as determined by a *k*-means cluster analysis (*k* = 2). (*b*) Community stability over time as measured by the site-level multivariate distances (blue lines) from their 2007–2013 centroid. The median multivariate distance across sites is shown as the black line with a 95% confidence region shown in grey. Blue lines depict individual site trajectories. The vertical dotted line represents the timing of the 2013 sea star wasting (SSW) event, and the 2014–2016 marine heatwave (MHW) is shaded in red. (*c–e*) Community taxonomic metrics showing (*c*) diversity, (*d*) richness and (*e*) evenness. Each point represents a single site and splines (λ = 0.05) were fitted across interannual means with 95% confidence intervals for sessile inverts and macroalgae (purple), mobile and conspicuous inverts (orange), kelps (green), and fishes (pink).

Beginning in 2014, outbreaks of purple sea urchins occurred throughout the region (figure 1), but the degree of kelp loss and the broader community consequences were spatially heterogeneous. Overall, the entire study region departed from a common multivariate (forested) state, which had persisted for at least 6 years (since the start of our 2007 data series) and drifted into a new multivariate cluster (figure 2*a*). This departure in community structure from the prior configuration coincided with the sea star wasting event, the marine heatwave, sea urchin outbreak, and decline in kelp (figure 1; electronic supplementary material, figure S2). These dynamics show a rapid destabilization in the regional structure of the community towards a new (at least within the 14-year data series), potentially stable, cluster in multivariate space (figure 2*a*,*b*).

At the regional level (i.e. across all sites), there were no substantial declines in taxonomic richness (number of species) for any group (fishes, kelp, mobile and conspicuous invertebrates, sessile invertebrates and macroalgae) resulting from the sea urchin outbreak and kelp forest declines (figure 2*d*). However, fish taxonomic richness exhibited gradual increases prior to the MHW, and a decline to levels early in the timeseries subsequent to the MHW (figure 2*d*). By contrast, fish taxonomic evenness was gradually declining prior to the MHW and eventually continued to decline after the MHW (figure 2*e*), leading to the overall decline in fish diversity (figure 2*c*). While evenness and diversity of the kelp assemblage did not change over the timeseries, evenness of both mobile and conspicuous invertebrates, and the sessile invertebrates and macroalgae declined subsequent to the MHW (figure 2*e*), leading to an overall decline in taxonomic diversity of these groups at the onset of the MHW (figure 2*c*). These results reflect a redistribution in the relative abundance (i.e. evenness) of species, rather than declines in the absolute number of species (i.e. richness).

The density of purple sea urchins significantly increased at all 24 sampled locations (figure 1; electronic supplementary material, figure S2; DF_1,3452_, *p* < 0.001). While the region-wide density of recorded sea urchins increased over 180-fold (from 0.06 ± 0.37 s.d. to 11.17 ± 19.58 s.d. urchins per m^2^) overall, the magnitude of kelp forest loss varied substantially among sites (electronic supplementary material, figure S2 and electronic supplementary material, figure S3). Following the sea urchin outbreak, mean kelp stipe density across all sites declined by 51%, from 1.92 stipes per m^2^ (± 1.71) to 0.94 stipes per m^2^ (± 1.72). As of the final year included in our analyses (2020), kelp density had declined by over 72% of the baseline average (from 1.92 ± 1.71 stipes per m^2^ to 0.53 ± 1.47 stipes per m^2^). However, there was a substantial degree of annual and site-level variation in kelp density (electronic supplementary material, figure S2), with some sites maintaining kelp densities comparable to baseline levels (electronic supplementary material, figure S3) despite the large increases in sea urchin density.

### (a) Spatial cohesion of community structure trajectory

All 24 sampled locations showed pronounced community structure shifts beginning around the year 2014 (electronic supplementary material, figure S4*a*, electronic supplementary material, table S3). Most sites were characterized by two temporally distinct clusters (from a *k*-means cluster analysis, electronic supplementary material, figure S5), with 2007–2013 falling into cluster 1, and 2014–2020 in cluster 2 (electronic supplementary material, figure S4*a*). In general, multivariate change was initially large but continued at a decreasing rate (figure 2*b*). However, the magnitude of community structure change varied by site. Stillwater DC, Macabee DC, Hopkins DC, and Bluefish DC showed the greatest shift in multivariate distance across years (indicated by the annual distance in nMDS space). These sites also continued to shift throughout the 2014–2020 period, as indicated by the large distance between years. However, Weston UC, Pescadero DC, Lone Tree and Bluefish DC all had very low interannual changes after the initial 2014 shift (electronic supplementary material, figure S4*a*). As of the final year included in our analyses (2020), community structure had not returned to its pre-2014 state for any site.

Temporal cohesion was strong among sites, indicating that community structure shifts followed similar trends through time, regardless of the degree of kelp loss (figure 2*b*; electronic supplementary material, figure S4*b*). However, there was variation in the relative strength of temporal cohesion, and geographically adjacent sites were not necessarily strongly cohesive (electronic supplementary material, figure S4B). For example, Bluefish UC and Bluefish DC are directly adjacent sites, but had the lowest pairwise cohesion score (electronic supplementary material, figure S4*b*), while other more geographically distant sites (e.g. Otter Pt DC versus Monastery UC) had relatively high (greater than 90%) cohesion.

### (b) Site trajectories and consequences of forest loss

**Figure 3.**
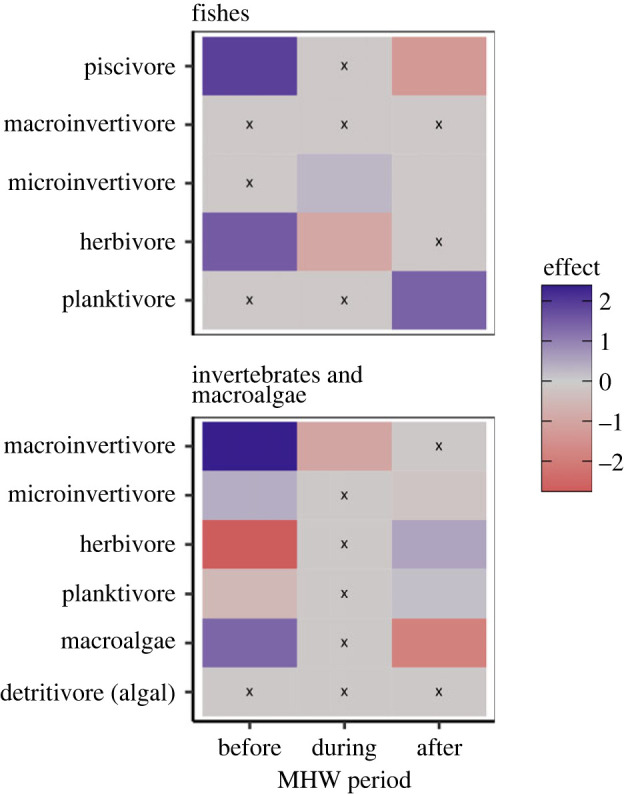
Contribution of interactions between marine heatwave period (before, 2007–2013; during, 2014–2016; after, 2017–2020) and taxonomic traits on variation in the relative abundance of fishes (top panel) and invertebrates and macroalgae (bottom panel). Colours depict the standardized coefficients for all period-trait interaction terms, based on the results of a multivariate abundance model. Coefficients were scaled to unit variance to make them visually comparable. Blue indicates a positive interaction between a given trait and period on species abundance, and red indicates a negative association. Black x's indicate interaction terms that were dropped from the multivariate model using a LASSO penalty.

Across the region, shifts in community structure were primarily explained by changes in the relative representation of macroalgae and invertebrates (figure 3). Declines in macroalgae explained the highest amount of taxonomic variation, and these declines initiated during the marine heatwave but were most pronounced after (figure 3). Declines in macro- and micro-invertivores also explained variation in the multivariate community structure. Only one piscivorous fish (black rockfish) was associated with variation in the relative abundance of taxa. Although densities of purple sea urchins increased at all sites, four persistent sites (Hopkins UC, Cannery UC, Cannery DC, Siren) maintained comparable densities of giant kelp before (2007–2013) versus after (2017–2020) the marine heatwave (electronic supplementary material, figure S3).

**Figure 4.**
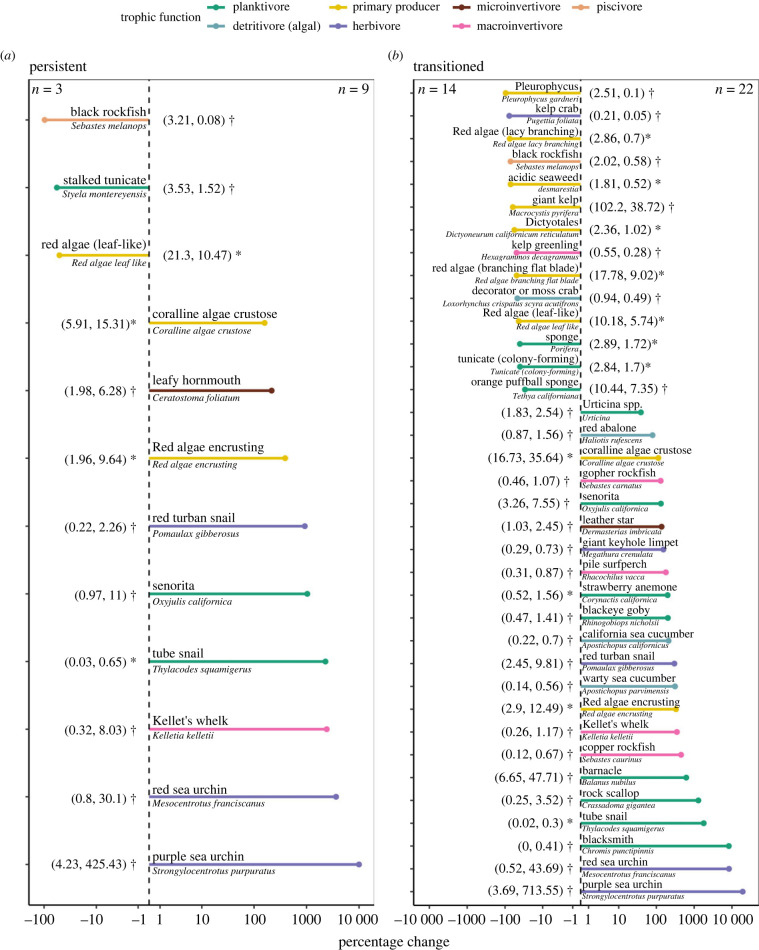
Changes in abundance of taxa that explained community shifts after (2014–2020) versus before (2007–2013) the sea urchin outbreak for (*a*) persistent and sites (*b*) transitioned. Only species that significantly contributed (as determined by simultaneous generalized linear models) to observed community structure differences between periods (after versus before) are shown. Each point connected with a horizontal line represents the percentage change in absolute abundance. Points to the left of the vertical dashed line indicate a decline in abundance and points to the right of the line indicate an increase in abundance. Line colour indicates the trophic function of a given species. Observed mean abundances before versus after the sea urchin outbreak are parenthetically included next to each species († = density, no. individuals per 60 m^2^ transect; * = percentage cover). The number of taxa that significantly contributed to community shifts are indicated in the upper left and right corners (left = number that declined, right = number that increased). Finally, sea star species were removed because of the wasting event that occurred in 2013.

The multivariate analyses revealed that taxonomic responses varied dramatically between persistent forests and sites that transitioned to barrens (figure 4). In the persistent forests, 12 species explained community structure shifts, but only three (black rockfish, stalked tunicate, red algae) declined in absolute abundance (figure 4*a*). However, in the sites that transitioned to barrens, 36 species explained community structure shifts, of which fourteen declined in absolute abundance (figure 4*b*). Primary producers (giant kelp, red algae, other brown algae) experienced the greatest decline in absolute abundance. There were also declines in planktivorous invertebrates (sponges, tunicates), one herbivore (kelp crab), and one detritivore (decorator crab). The transitioned sites were also characterized by a disproportionate (relative to the persistent forests) increase in many sessile invertebrate and algae species that are often dominant in sea urchin barrens, such as crustose coralline and encrusting red algae.

### (c) Predictors of kelp density and forest persistence

**Figure 5.**
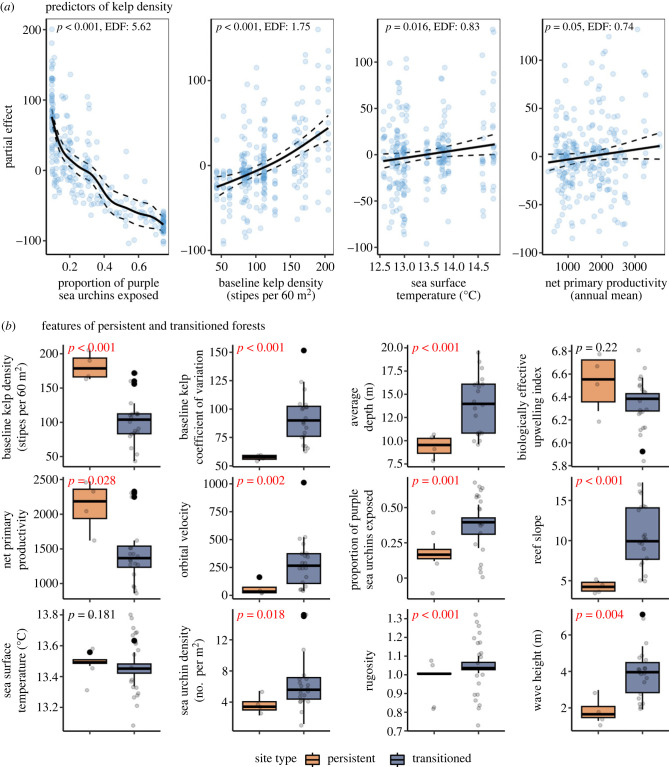
Environmental and ecological correlates of kelp forest dynamics. (*a*) Partial effects of significant model predictors on kelp density from a generalized additive model. Partial effects represent the smoothing term while holding all other variables constant. Solid black lines depict the shape of the relationship between each predictor and kelp density, and dashed lines represent 95% confidence intervals. Residuals are included as blue points. (*b*) Boxplots of each predictor variable for persistent and transitioned sites (see electronic supplementary material, figure S3). Persistent sites are those where kelp density was not significantly different (as determined by a *t*-test) from before (2007–2013) versus after (2017–2020) the marine heatwave and sea urchin outbreak. Transitioned sites are those where kelp density significantly declined (*p* < 0.05) during the 2017–2020 period. Also included are *p*-values from a *t*-test on a given variable between persistent and transitioned sites.

The generalized additive model (GAM) explained a large amount of variation in kelp stipe density as a function of sea urchin behaviour (proportion exposed), baseline kelp density, sea surface temperature and net primary productivity (*R*^2^ = 0.75, *n* = 259, *p* < 0.001; figure 5*a*; electronic supplementary material, table S4). Sea urchin behaviour and baseline kelp density were both strong nonlinear determinants of kelp density (EDF: 5.62 and 1.75, respectively). Sea surface temperature and net primary productivity were nearly linear, such that persistent forests were associated with slightly warmer temperatures and higher net primary productivity. However, although significant, sea surface temperature and net primary productivity were not as strongly associated with kelp density as sea urchin behaviour and baseline kelp density (electronic supplementary material, table S4).

Among the twelve predictors of forest persistence, ten were significantly different between persistent and transitioned forests (figure 5*b*). Persistent forests were characterized by having significantly higher baseline (average from 2007–2013) kelp density (*p* < 0.001) and net primary productivity (*p* = 0.028; figure 5*b*), and fewer exposed sea urchins. Persistent forests were also shallower (*p* < 0.001) with a more gradual reef slope (*p* < 0.001), and were more protected from wave exposure (i.e. lower wave height and lower orbital velocity; figure 5*b*; *p* = 0.005 and *p* = 0.002, respectively).

## 4. Discussion

This study reveals how environmental and ecological contexts shape ecosystem transition dynamics, and the consequences of community state shifts. As such, it contributes to a longstanding and growing recognition of how ecosystem dynamics reflect their ecological history (e.g.[47]). We detected a pronounced community destabilization event associated with the loss of a sea urchin predator (the sunflower sea star, *Pycnopodia helianthoides*) and an episodic marine heatwave event, prompting a sea urchin outbreak and kelp forest declines that shifted the entire region into a new multivariate configuration. This shift in community structure was primarily explained by changes in the relative abundance of macroalgae and invertebrates, with no substantial decline in the number of species, but pronounced declines in species diversity. These results add to a growing body of literature surrounding structural and functional changes in coastal marine communities [28,48–50] by revealing how punctuated environmental and biotic stressors (such as an episodic marine heatwave, a reduction in the abundance of a foundation species, and loss of a key mesopredator) can initiate shifts in ecosystem states.

While kelp forest shifts are often triggered by sea urchin density and recruitment increases (e.g. [18–20,50]), our results show that the historic density and stability (i.e. low change in multivariate distance) of macroalgae, and shifts in sea urchin grazing behaviour, were the strongest predictors of forest persistence and transition, respectively. Sea urchin density dramatically increased at all long-term monitoring locations, but despite this increase, some sites continued to persist with kelp densities comparable to baseline (2007–2013) averages. These persistent forests had the highest initial kelp density preceding the urchin outbreak, and it is likely that they produced sufficient detrital (drift) material to support high densities of passively-grazing sea urchins, thereby resisting density-driven direct herbivory [51,52]. Our results show that sea urchin density alone is not a single determinant of kelp forest loss, but grazer behavioural responses and environmental conditions can interactively influence persistence and transition dynamics [53].

Coastal upwelling and wave disturbance are the predominant drivers of kelp productivity and turnover on both sides of the Monterey Peninsula [33]. However, the four persistent forests identified in our study were all located along the inner northern Monterey Peninsula, which is characteristically sheltered from wave exposure and is typically warmer, shallower, and has a more gradual reef slope than transitioned sites [34]. Although these environmental conditions might support less productivity of persistent forests, they appeared to facilitate enhanced kelp stability, as indicated by the higher average stipe densities and lower coefficient of variation. This can in-turn confer resistance to overgrazing by providing enough consumable biomass such that herbivory does not completely devoid these patches of kelp. However, because the environmental conditions at persistent forests are generally less favourable for productivity, they might be limited in their rate of recovery. As of the final year included in our analyses (2020), none of the study sites had recovered to pre-2014 kelp densities. Therefore, continued monitoring is required to evaluate mechanisms of recovery.

The regional cohesion of community structure trajectory between sites highlights the scale at which mechanisms that facilitate state shifts are ecologically meaningful. Prior to 2013, all monitoring sites were formerly expansive forests that shifted into mosaics following coast-wide physical and biotic perturbations that occurred over a much larger spatial scale. Although community structure trajectories between sites across these regional mosaics were highly cohesive through time, sites experienced variable community structure changes. The onset of community destabilization was nearly synchronous in time between sites (occurring around 2014), and all sites rapidly departed from long-standing configurations into new multivariate configurations. Moreover, most sites became much more dominated by species reflective of the alternative barren state (i.e. void of macroalgae and dominated by encrusting red and coralline algae) of the system [18,19].

The impact of kelp forest loss on species-level changes in abundance is of great concern for conservation. Although community structure shifts occurred throughout our study region, there were pronounced differences in taxonomic-level changes between persistent and transitioned forests. Persistent forests continued to support similar assemblages of taxa, while transitioned sites experienced a decline in the abundance of several taxa. Primary producers such as brown and red algae were most impacted in the transitioned sites, likely reflective of declines resulting from direct herbivory. Interestingly, transitioned sites were explained by a decline in only one herbivore (kelp crab) and one detritivore (decorator or moss crab). Other areas to the north of our study region, where kelp loss was over 90%, experienced pronounced declines in macroherbivore abundance, most notably abalone, which prompted the immediate closure of the recreational fishery [54]. In our study system, we hypothesize that the persistence of macroherbivores could result from detrital material that is produced in patches of persistent forests and exported to adjacent barrens. Finally, black rockfish was the only fish species that explained community shifts, but it declined in both persistent and transitioned forests, suggesting that declines were not directly related to patch-level forest loss. Overall, the species-level impacts of sea urchin grazing appeared to be most pronounced in primary producers.

There is a global interest in understanding the thresholds and stabilizing factors for both kelp forests and the alternative barrens state of the system [55–59]. Alternative stable states are defined by a single set of environmental conditions that can support more than one successional end-state or equilibrium point [6,7,60,61]. By contrast, phase-shifts are driven by persistent changes in the environment that shift community structure, but with only one state of attraction under a given set of environmental conditions [6]. In this study, the sudden destabilization of the community occurred synchronously with an episodic marine heatwave, but the environmental conditions have largely returned to a pre-2014 state (although 2019 and 2020 were slightly warmer years) [50]. Therefore, the marine heatwave, reduction in the abundance of a foundation species (kelp), and loss of a key mesopredator (*P. helianthoides*) constitute a suit of sudden perturbations that likely tipped the system into an alternative sea urchin ‘barrens’ state. This is evidenced by the failure of community structure to return to the pre-2013 basin of attraction, despite the dissipation of the marine heatwave and return of pre-2014 environmental conditions. However, several other biotic and environmental mechanisms can facilitate state shifts. Forward state shifts (from forests to sea urchin barrens) can result from spatially explicit and episodic sea urchin recruitment [18,20,60,61], reduction in the availability of drift [51], declines in predator abundance [62], or from severe storms that result in the rapid loss of kelp biomass [26]. Reverse state shifts (from barrens to forests) can result from sea urchin disease epidemics [63,64] and from severe storms that physically dislodge exposed sea urchins [26].

Sea urchin predators can serve as highly influential mechanisms of stability [13,62] and are frequently cited for their role in reversing sea urchin barrens to a kelp-dominated state [65,66]. Other studies have identified the sunflower sea star (*P. helianthoides*) as an important predator for maintaining stability [67,68], and indeed the collapse of this predator is consistent with the timing of observed community structure destabilization. However, in this system, the sunflower star had relatively low numbers [30] before the onset of the wasting event. Therefore, herbivore control could have resulted from behaviourally-mediated (i.e. nonconsumptive) responses, rather than through consumption, although more research is needed to understand these mechanistic interactions [69]. Moreover, Smith *et al*. [23,30] demonstrated the role of sea otters in maintaining remnant patches of kelp forests in this study system. Therefore, although kelp persistence is associated with preexisting environmental conditions, grazer suppression by sea otters can further facilitate the longevity of patch resistance, ultimately contributing to enhanced ecosystem resilience through the maintenance of kelp spore production sources.

This study provides support for the hypothesis that kelp forest community structure can persist in a stable state for decades, but that episodic physical (e.g. marine heatwaves) and biotic (loss of predators, sea urchin outbreaks) perturbations can rapidly shift the community to alternative stable states. Moreover, spatial heterogeneity in preexisting ecological (kelp density and stability) and environmental conditions can result in mosaics of forest persistence and transition. The failure of community structure to return to the pre-perturbed state after the episodic marine heatwave provides support for the existence of multiple stable states and suggests that departures from long-term community configurations may be difficult to reverse [70]. Finally, this study highlights that in systems with alternative stable states, the community effects of large-scale perturbations seem localized and context-dependent.

## Data Availability

The data that support the findings of this study are openly available in DataONE from: https://opc.dataone.org/view/doi:10.25494/P6/MLPA_kelpforest.4 [69]. All processing and analysis code is archived in Zenodo and available at https://doi.org/10.5281/zenodo.10475747 [70]. Additional metadata are provided in electronic supplementary material [71].
